# Evaluation of the Effect of Two Volatile Organic Compounds on Barley Pathogens

**DOI:** 10.3390/molecules21091124

**Published:** 2016-08-26

**Authors:** Amine Kaddes, Olivier Parisi, Chadi Berhal, Sofiene Ben Kaab, Marie-Laure Fauconnier, Bouzid Nasraoui, M. Haissam Jijakli, Sébastien Massart, Caroline De Clerck

**Affiliations:** 1 Integrated and Urban Plant Pathology Laboratory, Gembloux Agro-Bio Tech (GxABT), University of Liège, Passage des Déportés 2, Gembloux 5030, Belgium; amine.kaddes@doct.ulg.ac.be (A.K.); Olivier.Parisi@ulg.ac.be (O.P.); chadi.berhal@doct.ulg.ac.be (C.B.); sofiene.benkaab@doct.ulg.ac.be (S.B.K.); mh.jijakli@ulg.ac.be (M.H.J.); sebastien.massart@ulg.ac.be (S.M.); 2 General and Organic Chemistry Unit, Gembloux Agro-Bio Tech (GxABT), University of Liège, Passage des Déportés 2, Gembloux 5030, Belgium; Marie-Laure.Fauconnier@ulg.ac.be; 3 Laboratory of Phytopathology, National Agronomic Institute of Tunisia, University of Carthage, Tunis 1082, Tunisia; nasraouibouzid2012@gmail.com

**Keywords:** methyl prop-2-enoate, methyl propanoate, biocontrol, volatile organic compound

## Abstract

This study aimed to determine the effect of Volatile Organic Compounds (VOCs) on some pathogens, these VOCs were emitted during interactions of barley with *Fusarium culmorum* Schltdl and/or *Cochliobolus sativus* Shoemaker, two common root rot pathogens. Our work shows that two organic esters: methyl propanoate (MP) and methyl prop-2-enoate (MA) significantly reduced the development of fungi in vitro. Additional tests showed that the esters significantly inhibited spore germination of these pathogens. The activity of these VOCs on a wide range of fungal and bacterial pathogens was also tested in vitro and showed inhibitory action. The effect of the VOCs on infected barley seeds also showed plantlets growing without disease symptoms. MA and MP seem to have potential value as alternative plant protection compounds against barley bioagressors.

## 1. Introduction

To compensate for their immobility, plants have developed mechanisms to interact with their biological environment. Among these, the emission of volatile organic compounds (VOCs) has attracted the interest of the scientific community since the 1980s [1,2,3,4,5]. According to their origin (flowers, fruits, leaves and roots) [1,2], VOCs have been shown to act as a language between plants but also between plants and insects [1,6]. For example, VOCs emitted by flowers and fruits of many plants are known to attract pollinators and seed dispersers [7]. Some insects such as bees (*Apis mellifera* Linnaeus) are even able to discriminate plant cultivars emitting the same volatile compounds, but at different levels [8].

In addition to their properties [9] (high vapor pressure, low molecular weight, lipophilic nature), VOCs can also activate direct and indirect plant defenses [3]. A plant attacked by an herbivore can indeed emit volatile compounds that will attract enemies of the phytophagous insect (parasitic wasps, flies and predatory mites), protecting itself from additional damage. Moreover, studies have shown that healthy plants were able to recognize VOCs emitted by a plant infected by a pathogen or attacked by an enemy in the neighborhood, and to react by emitting defense volatile compounds [7]. The signals emitted by plants can be very specific, according to the kind of noxious organism they meet. As an example, VOCs emitted from the leaves of the Lima bean infested with *Tetranychus urticae* Koch induced the expression of genes encoding pathogenesis-related proteins, lipoxygenase (LOX), ammonialyase phenylalanine (PAL) and farnesyl pyrophosphate synthase (FPS) in neighboring bean plants, activating the defenses of these plants [3,10].

More recently, researches have highlighted that some VOCs could have antifungal effects [11,12]. VOCs emitted by the fungus *Ceratocystis fimbriata* Ellis & Halst (butyl acetate, ethyl acetate and ethanol among others) for example, have shown to inhibit the mycelium growth of *Monilinia fructicola* G. winter honey and *Penicillium digitatum* Sacc, the causative agents of post-harvest diseases in peach and citrus, respectively [13]. In a similar way, VOCs emitted by *Fusarium oxysporum* Schltdl strain CanR-46 have been shown to inhibit the growth of 14 fungi of economic importance [14].

VOCs are thus considered as a promising tool in the development of new control methods [15]. These could consist in a direct application of the synthesized volatiles to the field or in the elicitation of volatile production to activate the induced defense systems of plants but should be seen as a complementary biotechnological tool in systems of integrated pest management [3,15].

Even if studies about below-ground emissions of VOCs are still much rarer, it was shown that these emissions were involved in interactions with rhizosphere organisms [2,16,17]. However, little is known about the systemic induction of root defenses [4,18] and there are very few examples of researches studying the modification of the blend of volatiles emitted by plant roots following a pathogen attack [19,20]. Fiers et al., have shown that the VOCs of barley roots infected by two pathogens (*Fusarium culmorum* and *Cochliobolus sativus*) are different from the ones emitted by healthy barley roots [3]. They also showed that the VOCs emitted by the infected barley roots have an inhibition effect on the growth of these pathogens.

Moreover, they have identified 23 and 21 compound emitted de novo during the interaction of barley roots with *F. culmorum* and *C. sativus*, respectively. In this study, we evaluate the fungicidal potential of the five more abundant de novo compounds that were commercially available, in the aim of giving tools for the development of a new biocontrol method against barley pathogens in the future.

## 2. Results

### 2.1. Evaluation of the Effect of Five Volatiles Organic Compounds on the Growth of Fusarium culmorum and Cochliobolus sativus

The effect of five VOCs on the growth of *F. culmorum* and *C. sativus* was evaluated at 25, 50, 100 and 500 μM. We have observed that the VOCs had limited effect on the growth of the fungi at low concentrations (25–50 μM). Indeed, the highest inhibition rates at these concentrations did not exceed 23% and 32% (for methyl prop-2-enoate), respectively after 240 h of incubation (see Tables S1 and S2).

**Figure 1:**
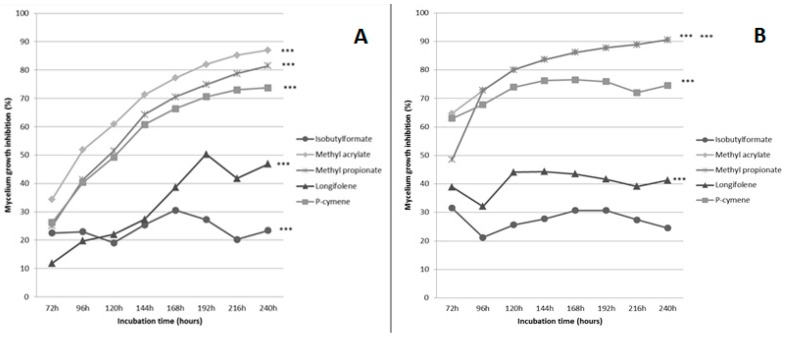
Growth inhibition of *F. culmorum* (**A**) and *C. sativus* (**B**) in presence of VOCs at 500 μM. Stars (***) show highly significant results.

At 100 μM, methyl prop-2-enoate was the only VOC showing a strong, statistically significant, growth inhibition of both fungi (83% for *F. culmorum* and 71% for *C. sativus*). At 500 μM, the influence of the five VOCs on the pathogens was clearer. Statistical analyses showed that the effect of all VOCs was highly significant on the growth rate of *F. culmorum* and that four VOCs (MA, MP, *p*-cymene and longifolene) had a highly significant effect on *C. sativus* (Figure 1).

The effect of MA at 500 μM was particularly marked, going up to 87% and 91% of growth inhibition for *F. culmorum* and *C. sativus*, respectively (Figure 1a,b). For MP also, growth inhibition was the highest at 500 μM going up to 81% and 91% for *F. culmorum* and *C. sativus*, respectively. *p*-Cymene also seemed to be efficient, but its effect was less marked (73% and 76%, respectively). Based on these results, MA and MP were selected to perform further tests.

### 2.2. Effect of Methyl Prop-2-Enoate and Methyl Propanoate on Spore Germination

After 2 h, a highly significant inhibition of *C. sativus*’s spores germination was already observed for MA and MP treated samples at 500 μM. Inhibition rates increased up to 69% and 57%, respectively compared to the untreated control. Similar results were observed with *F. culmorum* (Table 1). Six hours after the addition of the esters, the germination rates were increasing, probably due to the fact that the flask were opened several times to make measures and to the high volatility of the VOCs.

### 2.3. Evaluation of the Antifungal and/or Antibacterial Activity of Methyl Propanoate and Methyl Prop-2-Enoate on Several Pathogens

**Figure 2:**
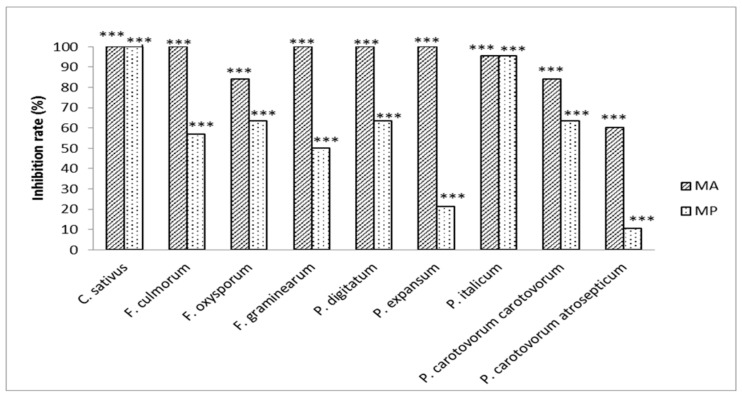
Effect of MA and MP (500 μM) on various pathogens after an incubation of 120 h for fungi and 12 h for bacteria. Stars (***) indicate highly significant differences with the control according to Dunnett’s test (*p* < 0.01).

The activity of MA and MP was then further analyzed on several plant pathogens. We observed that MA totally inhibited the growth of five out of the seven tested fungi after 120 h of incubation (Figure 2).

The growth of the two remaining ones was also highly reduced in the presence of the volatile compound (84% for *F. oxysporum* and 95% for *P. italicum*). MP effect was less pronounced, either on *P. expansum* (21.3%) or on *F. graminearum* (50%). The effect of the VOCs on bacteria was more contrasted but still statistically highly significant, with MA inhibition values ranging from 60% to 84% for *P. carotovorum atrosepticum* (PCA) and *P. carotovorum carotovorum* (PCC), respectively and being not higher than 63% in the case of MP used on PCC. The effect MP was particularly weak on PCA, with 10.6% of growth inhibition only.

### 2.4. Evaluation of the Protective Activity of Methyl Propanoate and Methyl Prop-2-Enoate on Infected Barley Seeds

In order to assess the efficacy of MA and MP to eliminate infections on the seeds, we observed the effect of both VOCs on artificially infected barley seeds (Table 2). All the uninfected seeds germinated and showed no disease symptoms on the leaves. After inoculation with *F. culmorum*, only 40% of the seeds germinated. The remaining ones did not germinated because of the development of the fungal mycelium. Among the germinated seeds, 83% showed symptoms on their first leaves. Regarding *C. sativus*, 47% of the infected seeds germinated and all the germinated seeds showed symptoms on their first leaves.

**Figure 3:**
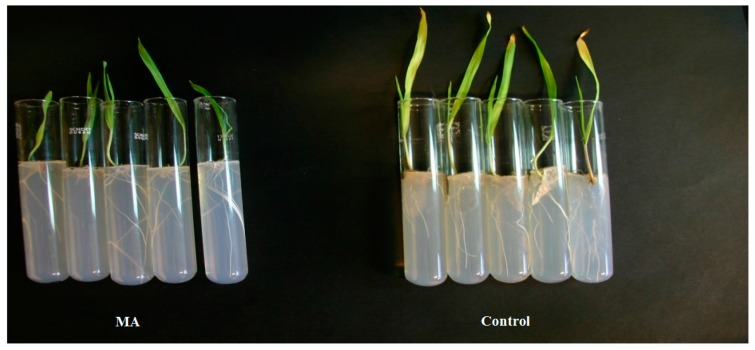
Effect of MA on seeds infected with *F. culmorum*. Untreated controls are on the right and the MA treated seeds are on the left.

**Figure 4:**
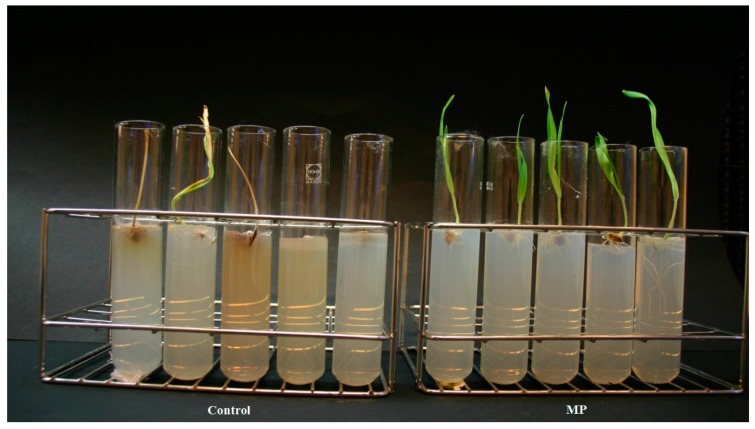
Effect of MP on seeds infected with *Cochliobolus sativus*. Untreated controls are on the right and the MP treated seeds are on the left.

After treatment with MA, 100% and 93% of the seeds infected by *F. culmorum* or *C. sativus*, respectively germinated and developed without showing any symptoms of the spot blotch disease (Figure 3 and Figure 4). Similar results were obtained after treatment with MP (Table 2). Statistical analysis showed that MA and MP significantly reduced (*p* < 0.05) symptoms development of both fungi on the first barley leaf. No phytotoxicity was observed on uninfected plantlets grown on medium containing the esters.

## 3. Discussion

The aim of this work was to study the potential antibacterial and/or antifungal effect of five VOCs produced de novo during the interaction between barley roots and two pathogenic fungi: *F. culmorum* and *C. sativus*. Among the studied VOCs, two organic esters (MA and MP) were shown to be particularly effective, inhibiting mycelium growth and spore germination of both pathogens. In addition, we showed that these VOCs were active against a broad range of pathogens.

Even if some repetitions should be made in order to improve the interpretation of the VOCs effects, the results obtained in vivo confirmed the in vitro results since a large part the seeds treated with the esters did not developed disease symptoms. The effect of MA is particularly significant. This is not surprising as strobilurins, natural compounds produced by fungi of the *Basidiomycota* phylum [21] and widely used as fungicides, are VOCs carrying a methyl prop-2-enoate radical [22]. The effect of strobilurins on *F. culmorum* was already assessed by [23], while their effects on *C. sativus* were shown in the study of Kunova et al. [21].

MP also had an effect on the growth and germination of the pathogens, even if it was less marked than for MA. This VOC is formed by esterification of propanoic acid with methanol. Propanoic acid is well-known as fungicide and bactericide, mostly used in stored grains [24]. It is also known to have in vitro fungicidal effect on several plant pathogens including *Aspergillus* sp., *Penicillium* sp. and *Fusarium* sp. [25,26,27]. In addition, another ester of this organic acid (2-methyl propanonate) was shown to be emitted by *Oidium* sp. and to be effective against *Pythium ultimum* Trow [28].

MA and MP were identified as being emitted only by barley roots infected by *C. sativus* [3]. Since the origin (either fungus or plant) of these compounds remains unknown, their action on pathogens can lead us to raise several hypotheses regarding their roles in natural environment.

Studies have shown that VOCs can be emitted by fungi, acting as germination self-inhibitors. These VOCs avoid the germination of the spores when their densities are too high (overcrowding) or during the dormancy period ahead after the spores dispersion. This is for example the case of 1-Octen-3-ol for *Penicillium paneum* Frisvad and *Aspergillus bisporus* Kwon-Chung & Fennell [29,30]. Fungi have also been shown to produce fungicidal compounds that would give them an advantage in food competition in their natural environment. This is the case of strobilurins and oudemansins produced by species of *Strobilurus* and *Oudemansiella* genera [31]. This is also the case of the blend of volatiles emitted by a fungal endophyte of *Orchidaceae* (*Phomopsis* sp.) [32]. However in this situation, one of the fungi should have been less affected than the other by the VOCs, which is not the case in our study.

Another possibility is that MA and MP are emitted by the plant in response to the pathogen infection, as a defense system against it. Rodriguez et al. observed that VOCs emitted by bean cultivars resistant to a species of *Colletotrichum* had inhibition effects on the growth of the pathogen [33]. In the same way, several plant species have been shown to synthesize and emit terpenoids, known to be defense compounds, in response to an infection by fungal pathogens [34,35,36].

## 4. Materials and Methods

### 4.1. Biological Material

The fungal and bacterial species used throughout this study, as well as their culture conditions are detailed in Table 3. All cultures were done under a 16L:8D photoperiod.

### 4.2. Evaluation of the Effect of Five Volatiles Organic Compounds on the Growth of Fusarium culmorum and Cochliobolus sativus

Three organic esters (methyl propanoate (MP) (Sigma-Aldrich, Diegem, Belgium), methyl prop-2-enoate (MA) (Sigma-Aldrich), isobutylformate (Sigma-Aldrich)) and two terpenes (*p*-cymene (Sigma-Aldrich) and longifolene (Sigma-Aldrich)) that were identified in a previous study [3] as being de novo emitted by infected barley roots, were tested at 0, 25, 50, 100 and 500 μM. Each VOC was mixed with 40 mL of water agar (1% agar (Difco, Grenoble, France)) then poured in cell culture flasks of 600 mL (VWR, Leuven, Belgium). Water agar was chosen to be sure that no VOCs coming from the medium could influence the results.

After medium solidification, a 70 mm disk of a ten days old active culture of *F. culmorum* or of a three weeks old active culture of *C. sativus* was placed in the center of medium. The cell culture flasks were placed in a growth chamber under LED light (94 mmol photons/m^2^/s) with a 16L:8D photoperiod at 22 °C for 10 days. The radial growth (RG) of the fungi was determined by measuring the average of two perpendicular diameters.

The RG of the fungus was measured each 24 h with a graduated ruler until 240 h. A total of 15 flasks were used for each concentration and each fungus. The assay was replicated independently three times. Flasks were placed randomly in the culture chamber. The growth inhibition rate was calculated as follows:

(1) $$\text{Growth inhibition rate}=\frac{\text{RG control - RG treated sample}}{\text{RG control}}\times 100$$

Statistical analyses were performed with Minitab 17 Statistical Software (Minitab Inc., State College, PA, USA) [37]. The growth rates of each fungus in the presence of each VOC was determined through regression curves, and compared using the Kruskall-Wallis test, applying Bonferroni correction.

### 4.3. Effect of Methyl Prop-2-Enoate and Methyl Propanoate on Spore Germination

Conidial suspensions were prepared by pouring 1 or 4 mL of sterile water on PDA plates colonized by *C. sativus* or *F. culmorum*, respectively. The mycelium was gently scratched with a sterile scalpel and the water-conidia mix was filtered on a double layer of cheesecloth placed on a sterile funnel. The concentrations of the suspensions were determined by counting conidia on a Fuchs-Rosenthal counting chamber (Hecht Assistent, Sondheim/Rhön, Germany) according the manufacturer’s instructions. The concentrations were adjusted at 10^6^ conidia/mL for each fungus. In order to distribute the conidia evenly, 0.1% Tween 20 (Merck KGaA, Darmstadt, Germany) was added to each conidial suspension. One milliliter of the conidial suspension was diluted in 9 mL of PDB solution in the presence of 500 μM methyl prop-2-enoate or methyl propanoate. Test tubes were hermetically closed and incubated during 8 h. Every 2 h, 70 μL of the suspension were taken and the conidial germination was observed under an optical microscope (Primo star, Carl Zeiss, Germany) and compared to a non-treated control. Three observations of 100 spores were made for each pathogen and VOC.

Rate of spore germination inhibition was calculated according to the following formula [13,38]:

(2) $$\text{Rate of spore germination inhibition}=\frac{\text{Germination of control}\left(\text{\%}\right)\text{- germination of treated sample}\left(\text{\%}\right)}{\text{germination of control}\left(\text{\%}\right)}\times 100$$

Statistical analyses were performed with Minitab 17, using one way ANOVA and Dunnett’s multiple comparisons test.

### 4.4. Evaluation of the Antifungal and/or Antibacterial Activity of Methyl Propanoate and Methyl Prop-2-Enoate on Other Pathogens

The test was carried out on six fungi and two bacteria in 96 wells ELISA microplates (VWR) (Table 3) following the protocol described by Kouassi et al. [39]. Two hundred microliters of 10^6^ conidia/mL suspensions for fungi and 10^7^ cfu/mL suspensions for bacteria were prepared in pathogen’s appropriate medium (Table 3) and placed in a well, along with each organic ester at 500 μM. Eight replicates were made per object. Controls for each group of replicates were wells containing non inoculated medium, wells containing non inoculated medium and VOCs, wells containing medium inoculated with the fungi. Plates were sealed with a polyester sealing film (VWR) providing secure sealing around every well, minimizing evaporation and well-to-well contamination; and incubated at 23 °C in the dark. The effect of each organic ester on the growth of each pathogen was assessed by measuring the optical density (OD) of each well at 490 nm every 24 h during 120 h for fungi and every 2 h during 12 h for bacteria [39]. The efficiency rates were calculated as follows:

(3) $$\text{Efficiency rate}=\frac{\text{AV(D.O.}{\text{X}}^{\prime }\left(t\right)-\text{D.O.}{\text{X}}^{\prime }\left(t_{0}\right))-\text{AV(D.O.Hx}\left(t\right)-\text{D.O.Hx}\left(t_{0}\right))}{\text{AV(D.O.}{\text{X}}^{\prime }\left(t\right)-\text{D.O.}{\text{X}}^{\prime }\left(t_{0}\right))}\times 100$$

with: AV: Average; D.O.X′(t_0_): Optical density of the pathogen’s growth control (wells with inoculated medium) just after inoculation (t_0_); D.O.X′(t): Optical density of the pathogen’s growth control (wells with inoculated medium) after 120 h for fungi and 12 h for bacteria (t); D.O.Hx(t0): Optical density of the pathogen in association with the VOC just after inoculation (t_0_); D.O.Hx(t): Optical density of the pathogen in association with the VOC after 120 h for fungi and 12 h for bacteria (t).

Statistical analyses were performed with Minitab 17, using one way ANOVA and Dunnett’s multiple comparisons test.

### 4.5. Evaluation of the Protective Activity of Methyl Propanoate and Methyl Prop-2-Enoate on Infected Barley Seeds

Barley seeds (*Hordeum vulgare* L. cv. ‘Quench’) (Jorion, Kerkhove, Belgium) were surface sterilized according to the protocol of Fiers et al. [3]. Seeds were infected by submerging those 30 min in suspensions of 10^6^ conidia/mL of *F. culmorum* or *C. sativus*. The inoculated seeds were drained on paper filters, then placed individually in test tubes containing 40 mL water agar (10%) in which methyl prop-2-enoate or methyl propanoate were added to reach a concentration of 500 μM. Controls were realized by placing healthy and infected seeds on medium lacking the organic esters, and by placing healthy seeds on medium containing the organic esters at 500 μM. The test tubes were closed with cotton wool and placed vertically in a growth chamber under LED light (94 mmol photons/m^2^/s) with a 16L:8D photoperiod and a temperature of 22 ± 0.5 °C for 15 days. The experiment was repeated three times, five barley seeds being analyzed by object in each repetition. After 21 days, the number of germinated seeds was counted and symptoms’ apparitions were observed on the plantlets. Statistical analyses were performed with Minitab 17, using a binary logistic regression.

## 5. Conclusions

In conclusion, we show that two organic esters (methyl prop-2-enoate and methyl propanoate) produced by barley roots infected by *C. sativus* have an in vitro effect on a broad range of pathogens, especially on *F. culmorum* and *C. sativus*. To our knowledge this is the first time that these VOCs are identified as having such effects. These VOCs can be promising tools in the control of barley pathogens. However, more work needs to be done in order to evaluate the true potential of the VOCs as bio-pesticides and to better understand the origin and mode of action of such compounds. Slow release formulations involving alginate have already been shown to assure an efficient and homogeneous dispersion of volatile compounds and could be successfully used to formulate these active compounds [40].

## Figures and Tables

**Table 1:** Inhibition of the spores’ germination in the presence of MA and MP.

Pathogen	VOC	Inhibition Rate of Spore Germination (%) at Given Time (h)		
		2	4	6
*Fusarium culmorum*	MA	55, 1 ***	65, 7 ***	53, 3 ***
	MP	61, 2 ***	62, 2 ***	41, 9 ***
*Cochliobolus sativus*	MA	69, 4 ***	69, 1 ***	65, 1 ***
	MP	57, 6 ***	59, 0 ***	41, 6 ***

Stars (***) indicate highly significant differences in comparison with the control, according to Dunnett’s test (*p* < 0.01).

**Table 2:** Effect of MA and MP (500 μM) on barley seeds germination.

Effect	G1	L1	L2	S1	S2	SNGM	
Seed infected by *F. culmorum*	6	6	3	5	3	9	
Seed infected by *C. sativus*	7	7	7	7	7	8	
Seed infected by *F. culmorum* + MA	15	13	12	0	0	0	*
Seed infected by *C. sativus* + MA	14	12	11	0	0	1	*
Seed infected by *F. culmorum* + MP	15	13	12	0	0	0	*
Seed infected by *C. sativus* + MP	14	11	11	0	0	1	*

G1: number of germinated seeds (/15). L1: number of seedlings with emergence of 1 leaf. L2: number of seedlings with emergence of leaves. S1: number of seedlings with fungal symptoms on the 1st leaf. S: number of seedlings with fungal symptoms on the second leaf. SNGM: number of seeds not germinated because of the development of mycelium. Star (*) represent statistically significant results.

**Table 3:** Microorganisms used in this study.

Type of Organism	Species	Reference	Origin	Medium	Temperature	Concentration
Fungus	*Fusarium culmorum*	MUCL28166	Finland	PDA	20 °C	10^6^ sp/mL
Fungus	*Fusarium oxysporum*	MUCL38936	Belgium	PDA	20 °C	10^6^ sp/mL
Fungus	*Cochliobolus sativus*	MUCL46854	Georgia	PDA	20 °C	10^6^ sp/mL
Fungus	*Fusarium graminearum*	Pers. collection	Belgium	PDA	25 °C	10^6^ sp/mL
Fungus	*Penicillium italicum*	MUCL15608	United States	PDA	20 °C	10^6^ sp/mL
Fungus	*Penicillium digitatum*	CBS319.48	Baarn/Netherlands	PDA	20 °C	10^6^ sp/mL
Bacterium	*Pectobacterium carotovorum atrosepticum*	PCA 332	France	V8	28 °C	10^7^ cfu/mL
Bacterium	*Pectobacterium carotovorum carotovorum*	PCC 380	France	V8	28 °C	10^7^ cfu/mL

Two culture media were used to cultivate the strains: PDA (Scharlau, Spain) and V8 (for one liter: 100 mL of V8 juice, 200 mg of CaCO_3_, 20 g of agar). All these media were autoclaved at 120 °C for 20 min.

## Supplementary Materials

Supplementary materials can be accessed at: http://www.mdpi.com/1420-3049/21/9/1124/s1.
