# Supplementary material for: Evaluation of the Effect of Two Volatile Organic Compounds on Barley Pathogens
Source: Molecules. 2016 Aug 26;21(9):1124. doi: 10.3390/molecules21091124 (PMC6274465; doi:10.3390/molecules21091124)
Supplement: Supplementary file 1 [file molecules-21-01124-s001.pdf]

Supplementary Materials: Evaluation of the Effect of Two Volatile Organic Compounds on Barley Pathogens

Amine Kaddes, Olivier Parisi, Chadi Berhal, Sofiene Ben Kaab, Marie-Laure Fauconnier, Bouzid Nasraoui, M. Haissam Jijakli, Sébastien Massart and Caroline De Clerck

Table S1. Percentages of growth inhibition of *F. culmorum* in presence of VOCs at 25, 50 and 100 µM.

	**72 h**			**96 h**			**120h**			**144 h**		
	**25 µM**	**50 µM**	**100 µM**	**25 µM**	**50 µM**	**100 µM**	**25 µM**	**50 µM**	**100 µM**	**25 µM**	**50 µM**	**100 µM**
Isobutyl formate	0.26	10.50	26.77	0.23	7.13	22.07	2.02	7.55	18.09	5.35	7.48	16.23
longifolene	6.45	6.99	31.45	2.18	3.49	18.12	1.42	4.61	17.17	4.52	7.53	19.32
methylacrylate	19.15	33.52	65.07	15.12	29.77	69.07	6.75	19.84	72.02	11.64	24.21	76.89
methylpropionate	19.65	27.96	31.49	18.22	20.24	27.94	12.03	21.53	23.73	12.68	22.91	27.23
paracymene	12.77	16.87	39.52	11.07	14.12	32.63	9.41	13.58	29.48	8.56	11.67	22.96
	**168 h**			**192 h**			**216 h**			**240 h**		
	**25 µM**	**50 µM**	**100 µM**	**25 µM**	**50 µM**	**100 µM**	**25 µM**	**50 µM**	**100 µM**	**25 µM**	**50 µM**	**100 µM**
Isobutyl formate	6.40	5.89	13.88	5.06	5.20	11.52	5.27	4.56	3.73	6.04	3.25	5.56
longifolene	2.49	7.94	20.92	2.67	6.75	17.38	4.79	8.73	12.51	4.83	9.58	12.96
methylacrylate	11.11	22.64	78.61	9.42	21.11	80.40	15.18	25.52	82.07	19.82	27.23	83.18
methylpropionate	14.61	23.22	26.22	17.45	28.51	29.36	15.73	23.05	29.48	13.50	22.83	28.35
paracymene	12.10	16.54	24.31	10.96	15.63	23.45	13.01	17.47	25.85	13.76	18.74	25.87

Table S2. Percentages of growth inhibition of *C. sativus* in presence of VOCs at 25, 50 and 100 μM.

	**72 h**			**96 h**			**120h**			**144 h**		
	**25 μM**	**50 μM**	**100 μM**	**25 μM**	**50 μM**	**100 μM**	**25 μM**	**50 μM**	**100 μM**	**25 μM**	**50 μM**	**100 μM**
Isobutyl formate	0.43	13.92	16.67	0.71	12.85	14.18	5.54	13.99	13.12	7.56	11.84	15.62
longifolene	5.02	5.09	18.79	6.13	12.56	17.74	8.91	15.13	19.11	11.62	12.42	20.77
methylacrylate	23.43	42.79	62.15	16.98	37.26	64.15	20.58	37.09	65.24	21.24	34.45	67.39
methylpropionate	7.19	19.69	37.19	7.48	15.96	38.40	6.88	15.79	34.41	10.29	18.87	32.08
paracymene	3.62	18.10	25.57	3.76	14.85	21.80	2.83	14.13	21.66	0.68	14.62	19.67
	**168 h**			**192 h**			**216 h**			**240 h**		
	**25 μM**	**50 μM**	**100 μM**	**25 μM**	**50 μM**	**100 μM**	**25 μM**	**50 μM**	**100 μM**	**25 μM**	**50 μM**	**100 μM**
Isobutyl formate	8.24	11.58	14.70	9.46	11.78	14.48	8.19	10.58	12.12	4.79	7.88	8.96
longifolene	8.40	12.71	21.53	9.33	12.19	21.20	6.83	10.69	16.63	10.52	10.51	15.94
methylacrylate	22.22	35.69	70.00	19.11	31.46	69.68	23.04	32.95	70.74	20.52	32.31	70.96
methylpropionate	17.01	25.17	35.68	17.55	26.08	36.30	20.28	29.14	37.57	14.94	24.80	34.18
paracymene	2.36	17.73	21.39	2.63	17.49	20.86	4.37	21.48	24.52	5.36	20.04	25.83
